# Atmospheric pressure predicts probability of departure for migratory songbirds

**DOI:** 10.1186/s40462-022-00356-z

**Published:** 2023-05-01

**Authors:** Nathan W. Cooper, Bryant C. Dossman, Lucas E. Berrigan, J. Morgan Brown, Dominic A. Cormier, Camille Bégin-Marchand, Amanda D. Rodewald, Philip D. Taylor, Junior A. Tremblay, Peter P. Marra

**Affiliations:** 1 Migratory Bird Center, Smithsonian’s National Zoo and Conservation Biology Institute, MRC 5503, 3001 Connecticut Ave. NW, Washington, DC 20013 USA; 2 Department of Biology and McCourt School of Public Policy, Georgetown University, 37th and O Streets NW, Washington, DC 20057 USA; 3 Cornell Lab of Ornithology and Department of Natural Resources and the Environment, Cornell University, 159 Sapsucker Woods Rd, Ithaca, NY 14850 USA; 4 Department of Biology, Acadia University, 33 Westwood Avenue, Wolfville, NS B4P 2R6 Canada; 5 Motus Wildlife Tracking System, Birds Canada, Port Rowan, ON N0E 1M0 Canada; 6 Wildlife Conservation Society Canada, 169 Titanium Way, Whitehorse, YT Y1A 0E9 Canada; 7 Wildlife Research Division, Environment and Climate Change Canada, 1550 Av. D’Estimauville, Québec, QC G1J 0C3 Canada

**Keywords:** Atmospheric pressure, Cloud cover, Departure probability, Migration, Precipitation, Weather, Wind profit, Wintering

## Abstract

**Background:**

Weather can have both delayed and immediate impacts on animal populations, and species have evolved behavioral adaptions to respond to weather conditions. Weather has long been hypothesized to affect the timing and intensity of avian migration, and radar studies have demonstrated strong correlations between weather and broad-scale migration patterns. How weather affects individual decisions about the initiation of migratory flights, particularly at the beginning of migration, remains uncertain.

**Methods:**

Here, we combine automated radio telemetry data from four species of songbirds collected at five breeding and wintering sites in North America with hourly weather data from a global weather model. We use these data to determine how wind profit, atmospheric pressure, precipitation, and cloud cover affect probability of departure from breeding and wintering sites.

**Results:**

We found that the probability of departure was related to changes in atmospheric pressure, almost completely regardless of species, season, or location. Individuals were more likely to depart on nights when atmospheric pressure had been rising over the past 24 h, which is predictive of fair weather over the next several days. By contrast, wind profit, precipitation, and cloud cover were each only informative predictors of departure probability in a single species.

**Conclusions:**

Our results suggest that individual birds actively use weather information to inform decision-making regarding the initiation of departure from the breeding and wintering grounds. We propose that birds likely choose which date to depart on migration in a hierarchical fashion with weather not influencing decision-making until after the departure window has already been narrowed down by other ultimate and proximate factors.

**Supplementary Information:**

The online version contains supplementary material available at 10.1186/s40462-022-00356-z.

## Background

Weather is a ubiquitous factor in the daily lives of animals, and it can have both immediate and long-term impacts on reproduction [1–3] and survival [4–6]. To appropriately respond to weather conditions, animals have evolved a diverse array of behavioral adaptions [reviewed by 7]. In response to poor weather conditions, animals may seek shelter [8], move to lower elevations [9], alter the timing of daily activity patterns [10–12], change foraging strategies [13, 14], delay breeding [15, 16], or delay emergence from hibernation [17]. Because they spend so much of their lives in the aerosphere, flying animals such as birds are likely particularly impacted by atmospheric conditions, and weather has long been thought to alter avian behavior, including seasonal migration [18–21].

Migration phenology is ultimately controlled by endogenous time-keeping mechanisms [22, 23], but it can be proximately modified by intrinsic and extrinsic factors including sex, age, body condition, and weather [24–26]. Presumably these factors act in a hierarchical fashion, with sex and age narrowing the window for the beginning of migration prior to the effects of shorter-term changes in body condition and day-to-day variation in weather. Studies of broad-scale migration patterns using radar have demonstrated that migration intensity often increases on nights without precipitation, with supporting winds, when atmospheric pressure is rising, and when skies are clear [20, 27–30]. Precipitation immediately prior to departure increases thermoregulatory costs [31] and may inhibit insect activity and successful foraging [32–34], and during migration itself, precipitation can cause disorientation [27]. Wind can dramatically increase or decrease flight speed and the energetic costs of flight [35, 36]. Rising atmospheric pressure generally predicts warm temperatures and clear skies at synoptic spatial scales in the days ahead, and birds may use changes in pressure to predict weather in the near future [27, 28]. Finally, birds use celestial cues (i.e., sunset position, sunlight polarization patterns, stars) to orient and navigate [22], and they may therefore be more likely to depart on clear nights when such cues are visible [22, 37]. Radar is a powerful tool for describing broad-scale avian migration patterns, but it cannot be used to study the behavior of individual animals, and therefore how weather affects decision making at the level of the individual remains less certain.

Studying departure decisions made by individual birds has resulted in more variable conclusions about the role of weather when compared to radar studies. Many individual tracking studies have documented increased departure probability with supporting winds [38–40], while others have shown no effect of wind [41–43], variable effects of wind [44, 45], or increased departure probability with headwinds [26]. Similarly, although several studies have found that birds are more likely to depart on nights when atmospheric pressure is high and/or rising [26, 44, 46, 47], and with little cloud cover [37, 45, 48], others have found no or variable effects of pressure [37, 40] and cloud cover [44, 49]. By contrast, the effect of rainfall has been more consistent with several studies showing that departure probability increases on nights with little to no precipitation [26, 44, 45, 48].

How weather influences departure decision-making has been studied almost exclusively at stopover sites during migration (but see [26, 44]), which might explain the highly variable and often contrasting responses to weather previously observed. Birds choose to interrupt their migrations and use stopover sites to rest, recover, refuel, escape predators, and/or avoid inclement weather [50, 51]. Thus, even conspecifics at the same site may vary in terms of why they have chosen to stopover and also when and why they choose to depart [43, 51–53]. These differences could potentially result in individually divergent responses to the same weather patterns. Although individuals may also depart the breeding grounds for multiple reasons, studying individuals as they first depart the wintering and breeding grounds may control for some of this variation and could lead to a better understanding of how weather influences departure decision-making.

Here, we use an automated radio-telemetry system [54] to investigate how departure date is influenced by weather in four species of songbirds as they departed five wintering and breeding sites in North America. While controlling for possible effects of age, sex, and/or habitat, we tested six hypotheses about how weather might affect the probability of departure on any given night. The “supporting winds” model tested the hypothesis that wind speed and direction influence departure, with the prediction that birds would be more likely to depart on nights when winds were blowing towards their immediate destination at the time of departure. The “future weather” model tested the hypothesis that changes in atmospheric pressure influence departure date, with the prediction that departure probability would increase when pressure had risen over the 24 h leading up to departure. The “winds + future weather” model combines the previous two models. The “precipitation” model tested the hypothesis that precipitation over the 3 h leading up to departure influences departure date, with the prediction that birds would be more likely to depart on nights without precipitation. The “precipitation + future weather” model tested the hypothesis that both rainfall and atmospheric pressure during the lead up to departure affects departure probability, with the prediction that birds would be more likely to depart when it had not rained over the past 3 h and pressure trend was rising. Finally, the “celestial cues” model tested the hypothesis that birds need to be able to observe celestial cues (i.e., sunset position, sunlight polarization patterns, stars) on the night of departure, with the prediction that departure probability would increase with decreasing cloud cover at the time of departure.

In a previous study [55], we used automated radio-tracking data to determine whether individuals were initiating non-migratory regional movements or long-distance migratory flights upon departure. We found that American Redstarts (*Setophaga ruticilla*) departing Jamaica and Kirtland’s Warblers (*Setophaga kirtlandii*) departing The Bahamas and Michigan were most likely initiating long-distance migration upon departure. By contrast, Swainson’s Thrushes (*Catharus ustulatus*) and Blackpoll Warblers (*Setophaga striata*) breeding in Nova Scotia most likely made regional movements after departing and did not begin directed long-distance flights towards the wintering grounds for 10 days or more [56–58]. Detection data were too sparse to determine the post-departure movement type of Swainson’s Thrush departing Quebec. We hypothesized that this difference in post-departure movement type might affect how weather influences the probability of departure. Specifically, we predicted that weather would be more important for species initiating long-distance migration at departure, resulting in weaker effects of weather on departure probability in those populations first making regional movements.

## Methods

We studied departure phenology in four species at five study sites. On the wintering grounds, we studied Kirtland’s Warblers (hereafter Kirtland’s; *n* = 64) on Cat Island, The Bahamas (24.62°N, − 75.65°E), and American Redstarts (hereafter redstarts; *n* = 31) at the Font Hill Nature Preserve (18.03°N, − 77.94°E) in southwestern Jamaica. On the breeding grounds, we studied Kirtland’s breeding in the northern Lower Peninsula of Michigan (44.46°N, − 84.30°E; *n* = 46), and Swainson’s Thrush (hereafter Swainson’s; *n* = 23) breeding in Quebec, Canada at Forêt Montmorency (47.37°N, − 71.10°E). In Nova Scotia, we studied Swainson’s (*n* = 65) and Blackpoll Warblers (hereafter blackpolls; *n* = 49) breeding on Bon Portage Island (43.47°N, − 65.75°E) and Seal Island (43.40°N, − 66.02°E), which are located 2 km and 17.5 km from the mainland respectively [58]. See Additional file 1: Table S1 for detailed information on species included in the study.

### Capture, tagging, and automated telemetry

Using both passive netting and conspecific playback, we captured all individuals in standard avian mist nets. After capture, we aged (HY = hatch year, AHY = after hatch year, SY = second year, ASY = after second year) and sexed individuals when possible [59]. We then banded each individual with one aluminum U.S. Geological Survey band and up to three colored plastic bands and took standard morphological measurements. Next, we attached a coded radio-tag (NTQB2-1, NTQB2-2, NTQB3-2, or NTQB4-2; Lotek Wireless Inc., Newmarket, Canada) using a modified leg-loop harness [60]. Radio tags weighed between 0.29 and 1.0 g (all < 4.0% and most < 3.3% of body mass) and emitted individually identifiable signals at a single frequency with a pulse rate between 5.3 and 34.9 s ([56–58, 61, 62]; Additional file 1: Table S1).

To collect departure data, we used a local array of automated radio-telemetry stations (hereafter stations). Stations were part of the Motus Wildlife Tracking System [54], and each had 2–4 antennas that were able to detect and identify individually coded radio-tags at distances up to 10–15 km [54]. To determine departure date, we visually inspected data that were downloaded and processed using the “motus” package [63] in Program R [64]. For each tagged individual, we looked for patterns of signal strength that indicate departure [44, 52], and recorded the date and time that pattern was observed. We excluded any possible departures during morning twilight (*n* = 20) and between sunrise and sunset (*n* = 18) because such departures most likely represent individuals making local movements and not regional or long-distance movements [65]. For these individuals, detection data outside of the breeding or wintering site were not consistent with either regional or long-distance movements being made immediately after departure. In all cases, we suspect that the true departure event was not observed because these birds first moved locally and later departed from areas not covered by our local station arrays. This process left 280 departure events in our analysis (see Additional file 1: Table S1 for breakdown of sample size by species, sex, and age class).

### Data analysis

To determine how weather variables may have influenced departure date decisions, we first acquired weather data from the Copernicus Climate Change Service’s ERA5-Land [66] and ERA5 [67] datasets. The ERA5-Land dataset has a spatial resolution of 9 km, and we downloaded hourly total precipitation (mm/hr), surface pressure (Pa), and wind speed (m/s) and direction at 10 m above ground for the locations nearest to each study site. Cloud cover is not estimated in the ERA5-Land dataset, and so we downloaded hourly cloud cover data from the lower spatial resolution (31 km) ERA5 dataset. After downloading data, we created the following variables: wind profit (i.e., signed magnitude of wind speed relative to movement direction) during the hour of departure (m/s), 24-h pressure trend (Pa/hr), total precipitation (mm) over the 3 h leading up to departure, and proportional cloud cover during the hour of departure. For the 24-h pressure trend, we first used a linear regression to determine if the slope differed significantly from zero (*P* < 0.05), and then retained all significant slopes and recorded non-significant slopes as zero. We estimated wind profit [68] using wind speed and direction from the hour of departure and the estimated bearing each individual travelled as they departed the breeding or wintering grounds. Departure bearings were calculated for each individual that had multiple detections at different stations on the night of departure using package “swfscMisc” [69] in Program R [64] with two exceptions due to lack of station coverage in the vicinity of the study sites. For Kirtland’s in spring, there were no individuals detected at multiple stations on the night of departure, and so we used the first detections on the mainland United States to calculate the bearings, but only if they occurred within 2 days of departure. Redstarts had no detections within the first two days after departure, but all first detections on the mainland United States were in Florida. We therefore used the average bearing (340°; Range = 336–344°) between Jamaica and detections in Florida for all departing redstarts following Dossman et al. [70]. For individuals with no detections within the chosen time frames, we used the mean bearing for conspecifics at each study site.

To determine if weather variables predicted departure date for each species and location, we used Cox proportional hazards models [71]. These models estimate the hazard rate in relation to time-dependent and time-independent variables. In context of our analysis, the hazard rate can be interpreted as the daily probability of departure where hazard rates above one indicate increased probability and hazard rates below one indicate decreased probability [39]. We included weather from the hour of departure (wind profit, cloud cover), the 3-h leading up to departure (total precipitation), or the 24-h leading up to departure (pressure trend) as time-dependent variables in the models. These values were compared to the weather on all other days of the departure period starting at the same time of night as the eventual departure. The departure period was determined independently for each species and location by including each day starting with the three days prior to the earliest departure and ending with the departure event (Additional file 1: Table S1). Except when they could not be accurately determined in the field, we also included age and sex as time-independent covariates in models, but only if significant effects were found in separate ANOVA analyses of intra-specific variation in departure date (see Results). Habitat type (mangrove vs. second-growth scrub) was included in all redstart models because of its well-documented effects on body condition and departure date [24, 25, 72, 73]. Because of small sample size within age, sex, and habitat groups and to limit the complexity of the model set, we only tested for interaction effects between weather variables and age, sex, or habitat in the top models for each population. For all models, we determined whether the proportional hazards assumption, which assumes that the relative hazard is constant over time at different levels of the predictor, was met for each predictor variable. If any variable violated the proportional hazards assumption, we added an interaction effect between time and that variable, and then checked for violations of the proportional hazards assumptions again [74]. All Cox proportional models were run using Program R [64] package “survival” [74] and figures were created using package “simPH” [75].

To limit model complexity and avoid potentially strong correlations between predictor variables (e.g., precipitation and cloud cover), we created a separate model for each hypothesis (see Introduction) and then compared these six models to a null model separately for each species. The null model included age, sex, and/or habitat, but only if the effects of these variables were identified as significant in ANOVA’s run prior to creation of the Cox proportional hazards models. To rank competing models, we calculated Akaike’s Information Criterion adjusted for small samples sizes (AIC_c_) using the “AICcmodavg” package [76] in Program R [64]. Models within 2 units of the top-ranked model were considered as potentially informative, unless they differed only by the addition of a single parameter and the maximum log-likelihoods were similar [77]. Such models were removed from the summary of supported models (Table 3) but are shown in the full model results (Additional file 2: Table S2). We interpreted individual predictors within the top model(s) as informative if the 95% confidence interval of the hazard ratio did not overlap one. Means are reported ± 1 S.E.

## Results

### Weather during the departure period

Weather varied both within and between the different breeding and wintering sites present in our study (Table 1). Most sites experienced a wide range of wind conditions, but redstarts in Jamaica and Swainson’s in Quebec rarely experienced strong supporting winds. Nights during the departure period rarely had more than trace amounts of precipitation (> 2.5 mm/h; [78]) in all locations except The Bahamas, which experienced more than trace amounts of precipitation on 12% of nights during the departure period. Birds in Jamaica and The Bahamas experienced roughly equal proportions of nights with falling and rising pressure, while the remaining species experienced predominantly falling or stable pressure trends. Finally, cloud cover varied greatly by site, with Jamaica rarely experiencing overcast nights (≥ 95% cloud cover) during the departure period, while the remaining sites more frequently experienced overcast nights (Table 1).

**Table 1:** Descriptive weather statistics for species and locations included in this study

Species	Atmospheric pressure % nights falling/stable/rising	Wind profit median ± IQR [range]	Precipitation % nights > trace (%)	Cloud cover median (IQR)—% of nights overcast
American Redstart (Jamaica—Spring)	41% / 7% / 52%	0.6 ± 2.0 [− 3.5 to 3.3]	1	0.2 (0.13–0.48)—6.9%
Kirtland's Warbler (Bahamas—Spring)	38% / 19% / 43%	3.8 ± 4.5 [− 3.9 to 9.2]	12	0.6 (0.27–0.95)—26.0%
Kirtland's Warbler (Michigan—Fall)	52% / 34% / 13%	− 1.3 ± 1.9 [− 4.3 to 4.5]	1	0.3 (0.03–0.85) – 20.1%
Swainson's Thrush (Quebec—Fall)	44% / 49% / 7%	− 0.5 ± 1.8 [− 4.3 to 2.0]	8	0.7 (0.08–0.98)—34.1%
Swainson's Thrush (Nova Scotia—Fall)	48% / 43% / 9%	0.2 ± 5.6 [− 9.2 to 9.0]	5	0.8 (0.05–1.00)—42.5%
Blackpoll Warbler (Nova Scotia—Fall)	46% / 44% / 10%	0.2 ± 4.7 [− 8.9 to 8.8]	2	0.7 (0.07–0.98) – 35.5.%

### Departure date

Within species, we found some evidence of age- and sex-based patterns of departure from the wintering grounds. In redstarts, males (May 3 ± 1.2 d) departed a few days before females (May 6 ± 1.4 d), but the effect was only nearly significant (Table 2). Older redstarts (May 3 ± 1.0 d) departed before younger birds (May 7 ± 2.3 d), and birds in mangrove (May 1 ± 1.9 d) departed before those in scrub (May 5 ± 1.1 d; *F*_1,27_ = 4.9, *P* = 0.046). In Kirtland’s, older (May 1 ± 1.2 d) and younger (May 2 ± 1.0 d) individuals departed on similar dates from The Bahamas, but males (April 30 ± 0.7 d) departed about a week before females (May 8 ± 1.4 d; Table 2). We found no other significant effects of age or sex on departure date from the wintering grounds (Table 2).

**Table 2:** Results from ANOVA of departure date by age and sex for each species and location except when age and/or sex were not known

Species	Age	Sex
American Redstart (Jamaica—Spring)	F_1,27_ = 4.89, P = 0.036	F_1,27_ = 3.15, P = 0.087
Kirtland's Warbler (Bahamas—Spring)	F_1,63_ = 0.46, P = 0.500	F_1,63_ = 22.82, P < 0.001
Kirtland's Warbler (Michigan—Fall)	F_2,42_ = 1.12, P = 0.335	F_1,42_ = 0.002, P = 0.961
Swainson's Thrush (Quebec—Fall)	–	–
Swainson's Thrush (Nova Scotia—Fall)	F_1,63_ = 8.76, P = 0.004	–
Blackpoll Warbler (Nova Scotia—Fall)	F_1,47_ = 21.61, P < 0.001	–

On the breeding grounds, we found some evidence for effects of age, but it varied by population. Hatch-year Swainson’s from Nova Scotia (Aug. 29 ± 1.2 d) departed a few days earlier than adults (Sep. 4 ± 1.5 d), and in blackpolls, hatch-year birds (Aug. 24 ± 1.0 d) also departed before adults (Sep. 3 ± 2.2 d). All other age and sex effects were either not significant or not tested for because age and sex data were unavailable (Table 2).

### Weather and departure probability

**Fig. 1:**
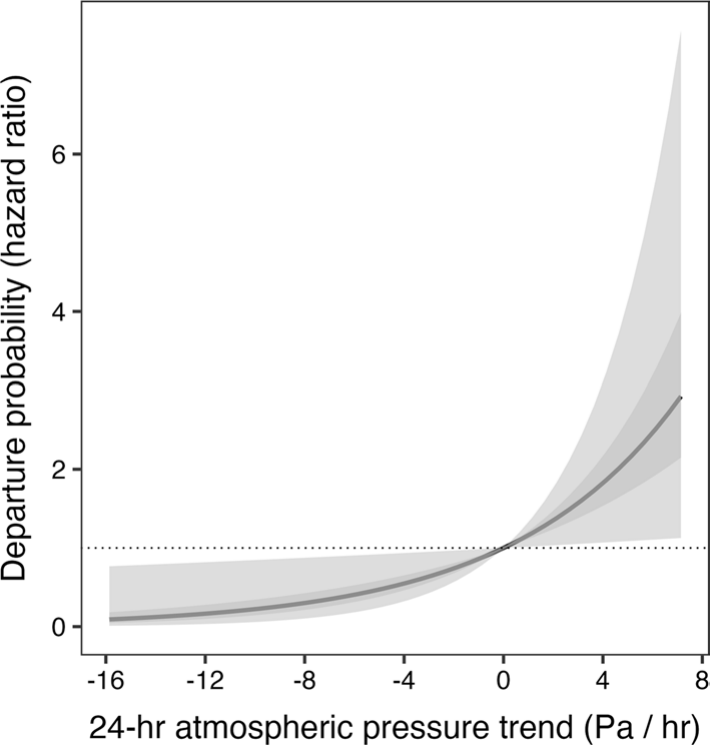
The effect of change in atmospheric pressure over 24 h (Pa/h) on the spring departure probability (hazard ratio) of American Redstarts (*Setophaga ruticilla*) wintering in Jamaica. Dark grey line represents the mean effect, and the 50% and 95% confidence intervals are shown in dark and light grey respectively

**Fig. 2:**
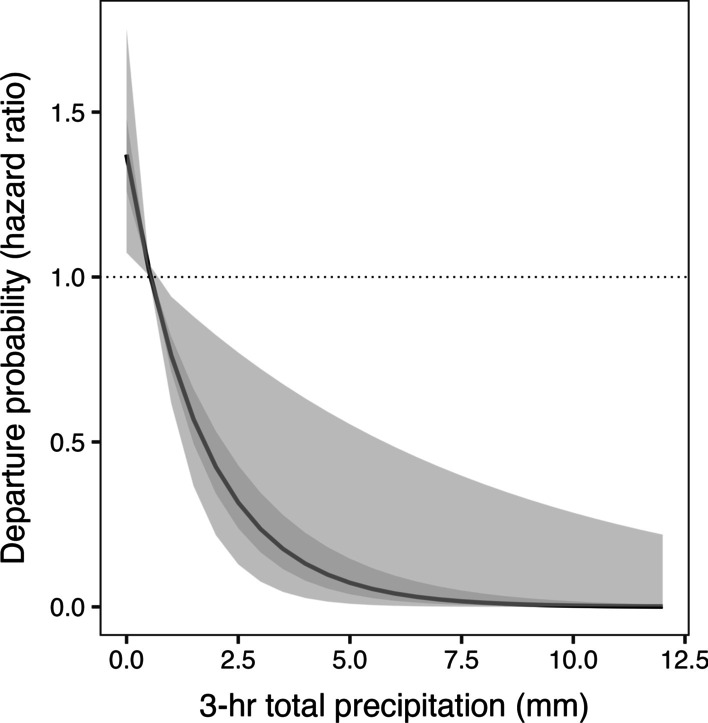
The effect of precipitation (mm) over the 3 h prior to departure on the departure probability (hazard ratio) of Kirtland’s Warblers (*Setophaga kirtlandii*) wintering in The Bahamas. Dark grey line represents the mean effect, and the 50% and 95% confidence intervals are shown in dark and light grey respectively

On the wintering grounds, model selection indicated strong support for the future weather model in redstarts and the precipitation + future weather model in Kirtland’s (Table 3). In redstarts, the probability of departure increased with rising pressure trend over the past 24 h (Fig. 1). Initial analysis of Kirtland’s models of wintering ground departure revealed that the effect of sex violated the proportional hazards assumption. We attempted to correct for this violation in several ways, but those efforts failed. After removing females (*n* = 13), the proportional hazards assumption was no longer violated and the precipitation + future weather model was the only model that received support. We found that males were more likely to depart when it had not rained in the three hours leading up to departure (Fig. 2) and when pressure was rising. Within the top-ranked models for departure from the wintering grounds, we found no important interactions between weather variables and age, sex, or habitat. We found little support for models with other weather variables, but the null model for redstarts, which contained the effects of age, sex, and habitat was ranked fairly high (Δ AICc = 3.06; Additional file 2: Table S2). This was expected because of the strong and well-documented effects of these variables on departure date in this population of redstarts [24, 72, 73, 79].

**Table 3:** Model statistics for top-ranked (< 2 Δ AICc) models for each species and location

	Δ AICc (AICcWt)	Weather Predictor 1	Weather Predictor 2
*American Redstart (Jamaica—Spring)*			
Future weather	0.00 (0.48)	HR_Pressure_ = 1.2 (1.01–1.33)*	–
*Kirtland's Warbler (Bahamas—Spring)*			
Precipitation + Future Weather	0.00 (0.99)	HR_Precip._ = 0.56 (0.35–0.89)*	HR_Pressure_ = 1.15 (1.05–1.25)*
*Kirtland's Warbler (Michigan—Fall)*			
Wind + Future weather	0.00 (0.56)	HR_Wind_ = 1.7 (1.32–2.18)*	HR_Pressure_ = 1.01 (0.99–2.18)
Wind	0.52 (0.44)	HR_Wind_ = 1.7 (1.33–2.16)*	–
*Swainson's Thrush (Quebec—Fall)*			
Future weather	0.00 (0.41)	HR_Pressure_ = 1.04 (1.01–1.07)*	–
*Swainson's Thrush (Nova Scotia—Fall)*			
Future weather	0.00 (0.50)	HR_Pressure_ = 1.03 (1.01–1.05)*	–
*Blackpoll Warbler (Nova Scotia—Fall)*			
Celestial cues	0.00 (0.56)	HR_Clouds_ = 0.16 (0.05–0.47)*	–
Future weather	1.66 (0.24)	HR_Pressure_ = 1.06 (1.02–1.11)*	–

Asterisks signify individual effects that were interpreted as informative based on the 95% confidence interval of the effect not overlapping with one. Full model structure and statistics are available in Additional file 2: Table S2

**Fig. 3:**
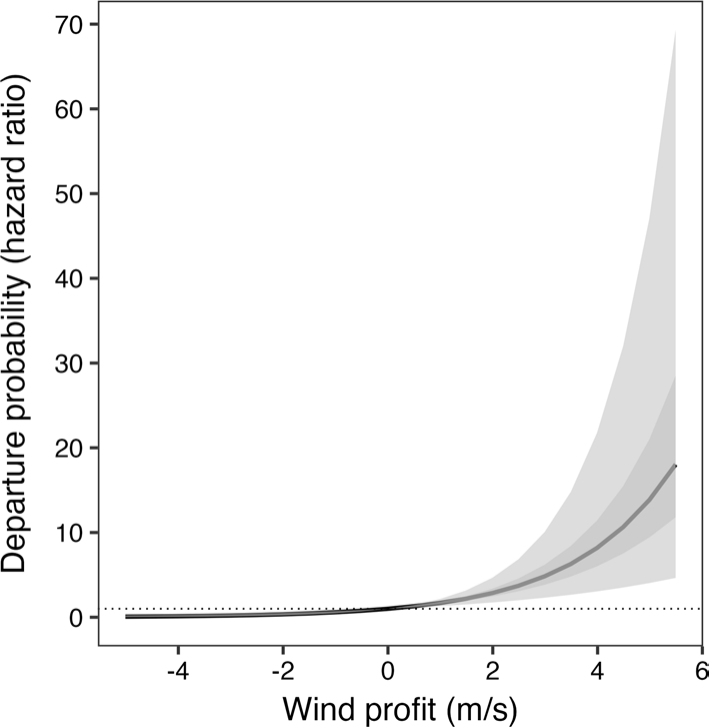
The effect of wind profit (m/s) at the hour of departure on the departure probability (hazard ratio) of Kirtland’s Warblers (*Setophaga kirtlandii*) breeding in Michigan, USA. Dark grey line represents the mean effect, and the 50% and 95% confidence intervals are shown in dark and light grey respectively

For departure from the breeding grounds, we found strong support for the future weather model in three populations and the celestial cues and wind + future weather models in one population each (Table 3). In Kirtland’s, the wind + future weather was the top-ranked model, and the probability of departure increased as wind profit increased (Fig. 3). Although pressure trend was included in the top-ranked model, the confidence interval of the effect slightly overlapped one, and it added little explanatory power compared to the wind only model. In both Swainson’s departing from Quebec and departing from Nova Scotia, the future weather model was the only supported model, with a positive effect of pressure trend on departure probability. In blackpolls, initial analysis revealed that it very rarely rained in the 3-h leading up to departure, which prevented proper estimation of the effects of precipitation and hindered model selection. After removing models with precipitation, the celestial cues model was the top-ranked model, with departure probability declining as cloud cover increased. However, the future weather model also received support, and we again found a positive effect of pressure trend on departure probability (Table 3). We found no informative interactions between weather variables and age or sex in the top models for departure from the breeding grounds. As predicted, the strength of the relationships between weather variables and departure probability was somewhat stronger in the species beginning migration compared to Swainson’s and blackpolls departing Nova Scotia, which carried out regional movements after departure. However, the 95% confidence intervals of the hazard rates often overlapped, limiting our ability to make strong inferences about the differences between species (Table 3).

## Discussion

Using a multi-species automated telemetry dataset collected at several breeding and wintering sites across North America, we found that weather influenced the probability of songbird departure in multiple species, seasons, and locations. On both the wintering and breeding grounds, individual variation in the probability of departure was often best predicted by changes in atmospheric pressure, with individuals more likely to depart when pressure had risen over the 24 h leading up to departure. High and rising atmospheric pressure is associated with warm temperatures, clear skies, and calm winds in the days ahead [27, 80] and observational studies have shown that many taxa, including insects [81, 82], reptiles [83], mammals [84], and birds [26, 44] can alter their behavior in response to changes in pressure. Moreover, manipulative studies of captive birds indicate that individuals can detect changes in atmospheric pressure and adjust their behavior in response [85, 86]. Thus, birds are able to detect changes in pressure, and by departing on nights when pressure has been rising, they likely improve the probability of a safe and efficient beginning to migration. Large storms during migration can kill songbirds by the thousands [87] and enhancing survival probability by migrating during good weather is likely critical because songbird mortality appears to be high during migration compared to the breeding and wintering grounds [88–90].

The departure probability of individuals making shorter-distance flights, like blackpolls and Swainson’s breeding in Nova Scotia, also increased with rising atmospheric pressure. Although confidence intervals overlapped, the effect of pressure on departure probability in these two populations was somewhat weaker than in redstarts and Kirtland’s departing the wintering grounds, but similar to that of Kirtland’s departing Michigan and Swainson’s departing Quebec. We do not fully understand the function of regional movements carried out by blackpolls and Swainson’s after departure, but they are likely related to prospecting, selection of high-quality post-breeding habitat, and/or the development of visual landmarks needed for navigation [56, 58]. The use of visual cues to orient, prospect for information, and/or select post-breeding habitat would presumably be enhanced during fair weather, and flying even short distances in fair weather has lower energetic costs than flying during inclement weather. Thus, even those individuals making relatively short-distance exploratory movements likely benefit from clear weather in the days immediately after departure.

Wind has the potential to more than double (or halve) flight speeds, but also strongly impacts the energetic costs of maintaining flight over long distances [29, 35], and birds should therefore benefit from initiating migration on nights with supporting winds. However, previous studies on wind selectivity have been inconclusive, with some studies finding that birds are more likely to depart with supporting winds [38–40], and others showing weak wind selectivity [41–43]. We only found an effect of wind on departure probability in Kirtland’s departing the breeding grounds. On their Michigan breeding grounds, Kirtland’s rarely experienced nights with supporting winds during the departure period (24% of nights), but 83% of individuals departed on nights with positive wind profit, indicating strong selectivity for supporting winds. Similar to Kirtland’s departing the breeding grounds, both redstarts and Kirtland’s departing the wintering grounds also primarily departed on nights with supporting winds (81% and 82% of individuals respectively), but supporting winds were the norm during the departure period (Jamaica = 67% of nights, The Bahamas = 87% of nights). Thus, in these two populations it may simply not have been necessary to delay departure to ensure a high probability of leaving on a night with supporting winds, and other factors such as precipitation or atmospheric pressure took precedence in departure decision making.

In contrast to those species beginning migration, we would not necessarily expect strong selectivity for supporting winds in populations carrying out regional movements after departure. Accordingly, we found no evidence that blackpolls and Swainson’s departing Nova Scotia preferred departing with winds that would have supported their generally northward flights to the mainland. However, these species may be more selective for winds once they actually begin long-distance migratory flights after finishing regional movements. We were unable to assess this possibility because the sparseness of the Motus Wildlife Tracking System outside of our breeding areas prevented us from determining the exact date, time, and location that individuals first making regional movements actually began long-distance migration. Nonetheless, our results are consistent with previous studies, which have found inconsistent levels of wind selectivity in songbirds (see above). Although flying with supporting winds has clear benefits in terms of flight speeds and energetics, the strength of wind selectivity may be species-, site-, and/or context-dependent. For example, several studies have suggested that delaying departure to wait for optimal wind conditions may not be adaptive if individuals are under strong time constraints related to the annual cycle [91–93].

Overall, we found little support for the idea that either precipitation or cloud cover influences departure. Precipitation only decreased departure probability in Kirtland’s departing The Bahamas, which contradicts previous radar studies that have generally found consistently negative effects of precipitation [26, 44, 45, 48]. However, this may result from the fact that there was rarely a significant amount of precipitation at most of our study sites, except in The Bahamas where greater than light rainfall was recorded on 12% of nights. Additionally, only the departure of blackpolls appeared to be affected by cloud cover, with departure probability decreasing on overcast nights, as has been found in some studies [37, 45, 48]. This indicates that birds generally do not need clear skies to depart their breeding and wintering grounds. The fact that birds use celestial cues to orient and navigate is without question, as it has been repeatedly documented in controlled experiments [94–96]. However, previous research suggests that birds may rely primarily on magnetic cues for navigation when other cues are unavailable, and birds may also integrate information from celestial cues on the nights prior to the actual night of departure [95, 97].

Collectively, the growing consensus from studies of departure at stopover, breeding, and wintering sites is that individual, site, species, and population differences are important contributors to variation in response to weather. The general consistency of our findings with regards to atmospheric pressure across different species, locations, and seasons compared to previous stopover studies suggests that studying departure from breeding and wintering sites may be helpful in determining the baseline rules of migration. However, even our results show that context can matter—Kirtland’s departure was affected by precipitation and pressure on the wintering grounds and primarily by wind on the breeding grounds. Although these and other differences may prevent broad generalizations about how birds respond to weather, individual-level patterns can potentially be integrated with broad-scale radar data to build population-level migration models that can advance our understanding and prediction of migration [30].

### Limitations and future directions

One of the major limitations in our study was our definition of the departure windows, which we based on the total range of departure dates for each species and location. Although departure phenology from breeding or wintering sites has only been rarely investigated, we know that factors unmeasured in the present study including habitat quality, food availability, body condition, and the timing of breeding (breeding season only) are all likely to influence departure schedules [24–26, 72]. Similar to the framework proposed by Müller et al. [98] for nocturnal departure time, we presume that decisions made about the date of departure are likely made hierarchically. Under such a framework, the departure window is ultimately controlled by the circannual rhythm, synchronized with the environment by photoperiod, and then proximately modified by various intrinsic and extrinsic factors. Given that fluctuations in habitat quality, food availability, and body condition occur on a longer time scale and have more delayed effects than weather, we propose that these, and probably other factors narrow the departure window prior to night-to-night decisions regarding immediate weather conditions. In this regard, Mitchell et al. [44] and Chmura et al. [26] both provide excellent examples of a more holistic approach, whereby they used reproductive timing data to predict relatively narrow windows of departure before beginning analysis of the effects of weather. Future study of departure would benefit from taking a similar approach by estimating individual differences in habitat quality, food availability, and body condition and predicting departure windows to use in subsequent weather analyses.

## Conclusions

Decisions about the date and time to depart breeding and wintering sites are a crucial part of the overall migratory strategy [99]. Although the importance of weather variables differed somewhat between populations, we found that changes in atmospheric pressure predicted departure probability in nearly all populations, wind profit was important in Kirtland’s departing the breeding grounds, and precipitation was important for Kirtland’s departing the wintering grounds. As predicted, we also found some evidence that the effects of weather on departure probability were stronger in species likely initiating long-distance migration compared to those first making regional movements. Although logistically challenging, progress in better understanding the ultimate and proximate factors that control departure timing may only come from season-long studies that collect data on many possible factors in the same individual birds (e.g., habitat quality, food availability, and body condition) and take into account the timing of other critical parts of the annual cycle such as breeding, parental care, and molt.

## Supplementary Information

**Additional file 1. Table S1.** Basic descriptive information for populations included in this study. Age was classified as second year (SY), after second year (ASY), hatch year (HY), or after hatch year (AHY) depending on the species.

**Additional file 2. Table S2.** Full model selection results from analysis of weather and departure probability.

## Data Availability

All detection data from the Motus Wildlife Tracking System (Projects 19, 49, 86, 109, 145) are publicly available at www.motus.org. Weather data were downloaded from the Copernicus Climate Data Store (https://cds.climate.copernicus.eu). All data necessary to reproduce the results are available at https://doi.org/10.25573/data.21514182.
